# Management Considerations for Tooth Extraction in a 70-Year-Old Survivor of the Hydrogen Bomb Test on the Lucky Dragon No. 5

**DOI:** 10.7759/cureus.77340

**Published:** 2025-01-12

**Authors:** Kento Nakamura, Yuto Morishita, Makoto Adachi, Yasuyuki Shibuya

**Affiliations:** 1 Oral and Maxillofacial Surgery, Nagoya City University Graduate School of Medical Sciences, Nagoya, JPN; 2 Oral and Maxillofacial Surgery, Nagoya Tokushukai General Hospital, Kasugai, JPN

**Keywords:** daigo fukuryu maru, hydrogen bomb, lucky dragon no. 5, radioactive fallout, tooth extraction

## Abstract

The Lucky Dragon No. 5 (Daigo Fukuryu Maru) was a Japanese tuna trawler exposed to radioactive fallout from the Castle Bravo hydrogen bomb test conducted by the United States on March 1, 1954, at Bikini Atoll in the Marshall Islands. All 23 crew members suffered acute radiation sickness as a result of the exposure.

In this paper, we present a case of the management of one of the Lucky Dragon No. 5 crew members who underwent tooth extraction 70 years after the nuclear test. Due to the patient's systemic exposure to radiation, various complications were anticipated following tooth extraction. However, systemic complications, post-extraction bleeding, local infection, and wound healing failure were not observed.

This article highlights the importance of careful systemic and local management during the perioperative period when treating radiation-exposed individuals, as well as the ongoing nuclear threat and the crucial role of oral surgeons in managing radiation-induced health issues and preparing for nuclear emergencies.

## Introduction

The Lucky Dragon No. 5 (Daigo Fukuryu Maru) was a Japanese tuna trawler that was exposed to radioactive fallout from the Castle Bravo hydrogen bomb test conducted by the United States on March 1, 1954, at Bikini Atoll in the Marshall Islands. While operating at sea, 160 km east of the hypocenter, the boat suddenly saw a flash of light westward and the sound of the explosion slowed down. Eventually, the "ash of death," or radioactive fallout, produced by the experiment fell on the Lucky Dragon No. 5 and all 23 crew members suffered injury from this nuclear test [1,2]. The crew returned to Japan after a two-week voyage.　

The systemic doses of the crew were evaluated from a minimum of 1.7 Gy to a maximum of 6.9 Gy, and all crew members were diagnosed with acute radiation sickness (radiation syndrome). Immediately after exposure, the crew had general malaise, loss of appetite, nausea, vomiting, diarrhea, and eye symptoms. After three days, erythema and blisters were observed on the limbs. Hair loss was seen after one week. After four to seven weeks, a decrease in WBC count and platelets was observed (WBCs = five patients with 2,000/μL or less, 13 patients with 3,000/μL or less, and five patients with 4,000/μL or less). After eight weeks, a recovery tendency of WBCs and platelet count was observed. One crew member died of multiple organ failure on day 207; the other 22 were discharged safely [3].

There are no reports of the tolerability of oral surgery among long-term survivors of atomic and hydrogen bomb survivors. In this paper, we describe the systemic and local management of the perioperative period when extracting the tooth of a crew member of Lucky Dragon No. 5, who was totally exposed to the explosion of a hydrogen bomb 70 years ago.

## Case presentation

This case was treated at the Department of Oral and Maxillofacial Surgery, Nagoya City University Hospital. Written informed consent was obtained from the patient for the publication of this case report and accompanying images. This study was conducted in accordance with the Declaration of Helsinki.

**Figure 1:**
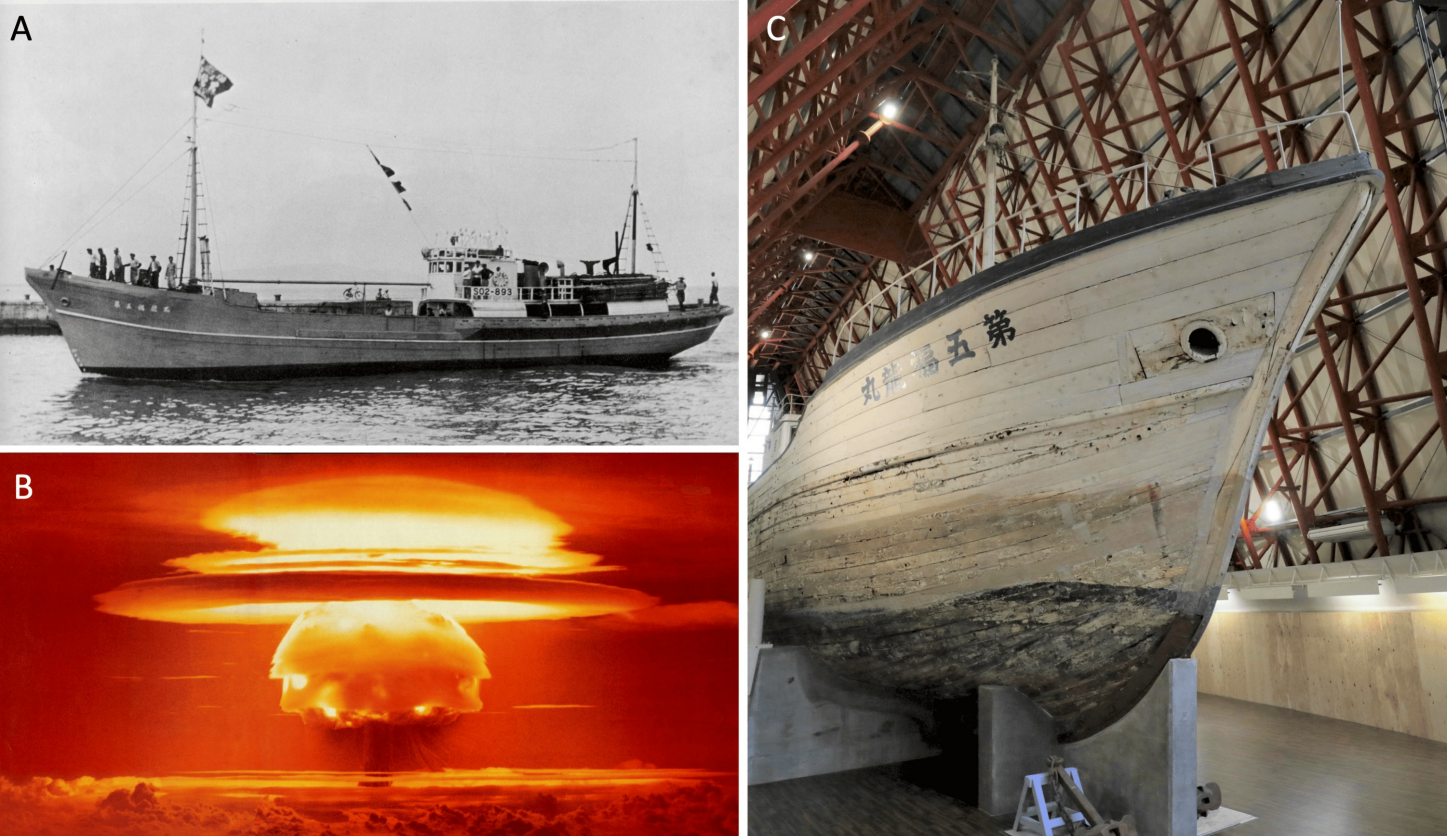
Lucky Dragon No. 5 (Daigo Fukuryu Maru) and Castle Bravo hydrogen bomb test (A) Lucky Dragon No. 5 (1954) during its operation as a deep-sea tuna fishing vessel. (B) Castle Bravo hydrogen bomb test conducted by the United States on March 1, 1954, at Bikini Atoll in the Marshall Islands. (C) The Lucky Dragon No. 5 is currently preserved and displayed at the Daigo Fukuryu Maru Exhibition Hall. Note: All historical photographs were provided by the Daigo Fukuryu Maru Foundation Inc. with permission for publication.

Lucky Dragon No. 5 was built in 1947 in Wakayama Prefecture as a bonito fishing boat but was later remodeled as a deep-sea tuna fishing vessel. The boat was exposed to radioactive fallout during the Castle Bravo hydrogen bomb test conducted by the United States on March 1, 1954, at Bikini Atoll in the Marshall Islands. The current Lucky Dragon No. 5 is on display at the Daigo Fukuryu Maru Exhibition Hall (Figures 1A-1C).

**Figure 2:**
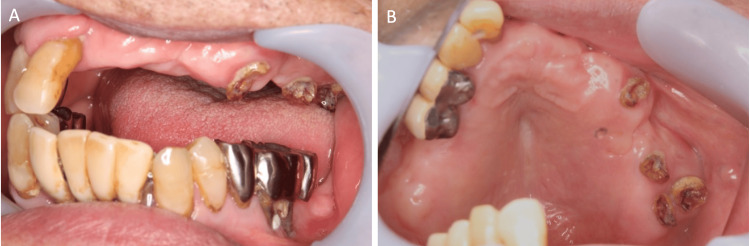
Intra-oral findings before tooth extraction (A) Preoperative intra-oral photograph showing residual roots of teeth #23, #25, and #26. The surrounding gingiva showed no notable inflammation. (B) Occlusal view of teeth #23, #25, and #26. The crowns of the teeth had collapsed and only residual roots remained.

Seventy years later, one of the survivors, an 86-year-old man, visited our department. He was a member of the crew of the Japanese tuna trawler Lucky Dragon No. 5 and needed to have teeth #23, #25, and #26 extracted because his dentist had determined that his teeth could not be preserved. Intra-oral findings at the first visit showed that teeth #23, #25, and #26 had collapsed crowns and were in a residual root state. The surrounding gingiva showed no notable inflammation (Figures 2A, 2B).

**Figure 3:**
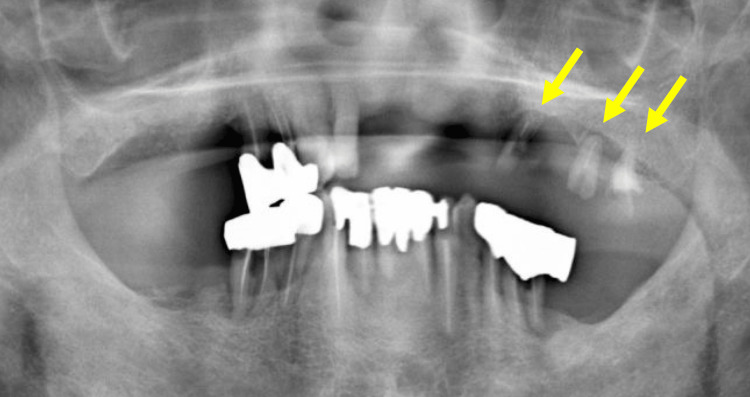
Panoramic radiograph taken at the first visit The panoramic radiograph revealed that teeth #23, #25, and #26 were residual roots. The surrounding alveolar bone was absorbed (arrow).

The panoramic radiograph revealed that teeth #23, #25, and #26 were residual roots and the surrounding alveolar bone was absorbed (Figure 3).

His medical history included hypertension, atrial fibrillation, diabetes, hepatitis C, and benign prostatic hyperplasia. For these clinical features, he was taking amlodipine, doxazosin, trichlormethiazide, dabigatran, and glimepiride. Laboratory examination revealed all hematological parameters within normal limits, with stable liver function tests. Glucose levels and HbA1c were slightly elevated, consistent with the patient's known diabetes. Renal function parameters were within the normal range (Table 1).

**Table 1:** Laboratory findings at preoperative examination

Parameter	Value	Reference Range
WBCs	5.5×103/μL	3.3-8.6×103/μL
RBCs	4.61×106/μL	4.35-5.55×106/μL
Hemoglobin	13.0 g/dL	13.7-16.8 g/dL
Hematocrit	38.8%	40.7-50.1%
Platelets	13.9×104/μL	15.8-34.8×104/μL
AST	25 U/L	13-30 U/L
ALT	10 U/L	10-42 U/L
Glucose	126.0 mg/dL	70-110 mg/dL
HbA1c (NGSP)	6.4%	4.6-6.2%
BUN	10.8 mg/dL	8.0-20 mg/dL
Creatinine	0.75 mg/dL	0.65-1.07 mg/dL
eGFR	74.0 mL/min/1.73m^2^	>45 mL/min/1.73m^2^

**Figure 4:**
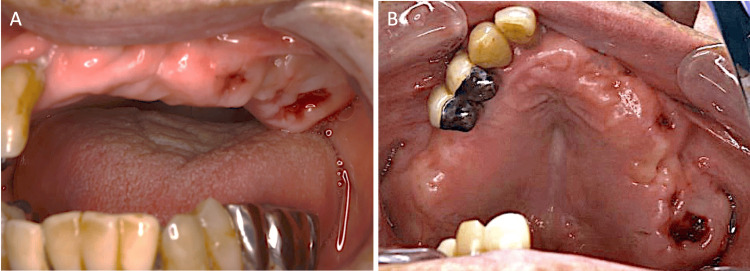
Intra-oral findings one week after tooth extraction (A) Postoperative intra-oral photograph showing healing of the extraction sockets. The gingiva was well adapted to the alveolar bone. (B) Occlusal view of the extraction sockets. The sockets were well healed and there was no evidence of infection or wound healing failure.

His physician also evaluated that he had no problem with his tolerance for tooth extraction under local anesthesia. Therefore, the individual underwent the extraction of teeth #23, #25, and #26 under local anesthesia. Given the patient's medical history of atrial fibrillation and anticoagulant therapy with dabigatran, a hemostatic splint was prepared preoperatively as a precautionary measure. The patient was fitted with the hemostatic splint after the tooth extraction. Dabigatran was not discontinued preoperatively. Amoxicillin 750 mg/day was administered from the day of tooth extraction. In the follow-up one week later, systemic complications, post-extraction bleeding, local infection, and wound healing failure were not observed (Figures 4A, 4B).

Clinical follow-up after three years showed no complications such as bone exposure, osteonecrosis, or septal bone separation.

## Discussion

We have performed tooth extraction on a survivor of the hydrogen bomb test at Bikini Atoll. Approximately 70 years have passed since the bombing, but no systemic or local complications were observed due to surgical intervention during tooth extraction under local anesthesia.

Atomic bombs are detonated using the energy generated when plutonium and uranium undergo nuclear fission. In contrast, a hydrogen bomb detonates by creating a high-temperature and high-pressure environment due to the initial explosion of an atomic bomb, which triggers a fusion reaction of hydrogen nuclei. Atomic bombs have an explosive power of 0.02 MT for Hiroshima-type atomic bombs, while hydrogen bombs have an explosive power of 15-17 MT [4]. 

The exposure dose would be 7 Gy (0.85 km from the center of the bomb) for a Hiroshima-type atomic bomb, while it would be 1.7-6.9 Gy (160 km from the center of the bomb) for a hydrogen bomb; also, hydrogen bombs are overwhelmingly more explosive than atomic bombs [5,6]. The exposure dose is also overwhelmingly higher. Members of the crew of Lucky Dragon No. 5, who were exposed during the hydrogen bomb experiment, have developed liver disease, mainly liver cirrhosis and liver cancer, following the bombing due to hepatitis virus infection caused by blood transfusions required after the bombing [7]. Hepatitis C was also found in this patient, but perioperative blood data showed no deterioration in liver function.

Radiation nuclides such as strontium-90 (90Sr), barium-140 (140Ba), caesium-141 (141Cs), cesium-137 (137Cs), and iodine-131 (131I) are released by the explosion of atomic bombs and hydrogen bombs, but all nuclides are released at a higher level for hydrogen bombs than for a Hiroshima-type atomic bomb [8,9]. Specifically, Sr-90 is considered to replace calcium in the bone, accumulate in the body, and continue to emit radiation for a long period. The effects of Sr-90 include bone loss, periosteal hardening and thickening, spot formation, and localized osteolytic changes in animal models [10]. The effects of internal exposure to radioactive strontium on the systemic skeleton include skeletal nutritional disorders that mainly affect the joints and tissues around the joints [11]. However, in this case, no skeletal disorder after tooth extraction was observed, and no abnormalities were found in the temporomandibular joint.

The passage discusses the ongoing threat of nuclear weapons and the importance of oral surgeons providing adequate medical care in a world where such threats persist. It highlights the devastating effects of atomic bombs used in World War II and the subsequent proliferation of nuclear weapons worldwide [12]. Despite global efforts toward nuclear disarmament, such as the Nuclear Non-Proliferation Treaty (NPT) and the Nuclear Weapons Ban Treaty (TPNW), the possession of nuclear power for peaceful purposes, such as nuclear power generation and X-ray equipment, continues to pose a nuclear threat. The accidents at the Chernobyl nuclear power plant in 1986 and the Fukushima Daiichi nuclear power plant in 2011 serve as stark reminders of the potential dangers associated with nuclear technology [13,14]. 

Oral surgeons play a crucial role in providing medical care to individuals affected by nuclear weapons and radiation exposure. They must be well-versed in the management of radiation-induced oral health complications, including radiation caries, salivary gland dysfunction, and osteoradionecrosis. Additionally, oral surgeons should be prepared to respond to potential nuclear emergencies and provide necessary dental care in such scenarios.

## Conclusions

The case of an 86-year-old patient, a former crew member of the Daigo Fukuryu Maru, provides important implications for the long-term dental management of patients exposed to radiation from nuclear weapons testing. Despite significant radiation exposure during the Castle Bravo hydrogen bomb test, the patient experienced no particular complications during or after tooth extraction. This experience helps us understand how radiation exposure can affect oral health and healing ability in the long term. This case also suggests the need to maintain detailed medical records and provide appropriate dental care to atomic bomb survivors, even decades after the accident. Furthermore, it illustrates the need for oral surgeons to consider not only current dental needs but also long-term health effects when treating patients with a history of radiation exposure.
